# The role of miR-433-3p in vascular calcification in type 2 diabetic patients: targeting WNT/β-Catenin and RANKL/RANK/OPG signaling pathways

**DOI:** 10.1007/s11033-023-08792-9

**Published:** 2023-09-20

**Authors:** Amira M. Elshamy, Yasser Mostafa Hafez, Mohamed A. E. Safa, Hoda A. Ibrahim, Mohamed Khalfallah, Fatma H. Rizk, Eman F. Eltabaa, Muhammad T. Abdel Ghafar, Marwa Mohamed Atef

**Affiliations:** 1 Medical Biochemistry Department, Faculty of Medicine, Tanta University, El Geesh Street, Tanta, 31511 Egypt; 2 Internal Medicine Department, Faculty of Medicine, Tanta University, Tanta, Egypt; 3 Cardiovascular Medicine Department, Faculty of Medicine, Tanta University, Tanta, Egypt; 4 Medical Physiology Department, Faculty of Medicine, Tanta University, Tanta, Egypt; 5 Clinical Pathology Department, Faculty of Medicine, Tanta University, Tanta, Egypt

**Keywords:** Vascular calcification, WNT/β-Catenin pathway, miR-433-3p, Dickkopf-1, RANKL, Osteoprotegerin

## Abstract

**Background:**

Vascular calcification (VC) is a major predictor of cardiovascular diseases that represent the principal cause of mortality among type-2 diabetic patients. Accumulating data suggest the vital role of some microRNAs on vascular calcification as an epigenetic regulator. Thus, we assessed herein, the role of serum miR-433-3p in vascular calcification in type-2 diabetic patients.

**Methods:**

Twenty healthy subjects (control group) and forty diabetic patients (20 without VC and 20 with VC) were involved in the study. miR-433-3p gene expression was measured. Runx2, Dickkopf-1 (DKK1), β-catenin, Receptor activator of nuclear factor kappa-B ligand (RANKL), and osteoprotegerin (OPG) levels in serum were assessed by ELISA technique.

**Results:**

Diabetes patients had significantly lower levels of miR-433-3p expression in comparison to the control group, with the lowest levels being found in diabetic patients with VC. Furthermore, Runx2, β-catenin, and RANKL levels were significantly increased with concomitant lower DKK1 and OPG levels detected in the two diabetic groups especially those with VC.

**Conclusion:**

Collectively, the study documented that down-regulation of miR-433-3p may contribute to the development of VC through activating WNT/β-Catenin and RANKL/RANK/OPG signaling pathways.

## Introduction

Vascular calcification (VC) is a major causative risk factor and a strong predictor of cardiovascular diseases (CVD), which remain the main cause of mortality in patients with type 2 diabetes [1]. It is defined as an ectopic abnormal deposit of calcium and phosphate minerals that leads to thickening and dysfunction of blood vessels, which is an important key factor contributing to CVD complications and mortality. Calcification of the vessel wall can occur in both the intimal and medial layers. However, it may occur also in heart valves [2].

Although VC occurs as a part of the normal aging process, diseases like diabetes, chronic kidney disease, and hypertension can also hasten their onset. [3]. In diabetic patients, VC commonly exists in the aortic wall, coronary arteries, and lower extremity arteries. Indeed, hyperglycemia has been reported to be one of the metabolic states promoting aortic valve fibrosis and calcification. [4].

Noteworthy, vascular calcification has complex and numerous pathogenic mechanisms. It results not only from high-calcium and phosphorous milieu but also happens as a result of oxidative stress, inflammatory cell infiltration, vascular smooth muscle cells (VSMCs) trans-differentiation endothelial dysfunction, osteogenesis, and matrix turnover [5, 6].

Interestingly, during VC, VSMCs exhibit a phenotypic switch into cells resembling osteoblasts (bone-forming cells). These cells undergo loss of smooth muscle biomarkers associated with increased expression of osteogenic biomarkers e.g. Runt-related transcription factor 2 (Runx2), osteocalcin, and osteopontin. Runx2 is a transcription factor that is essential for the differentiation of osteoblasts. VSMCs show upregulated Runx2 expression, which may be the reason for this cellular transdifferentiation [7, 8].

Runx2 is considered to be responsible for the osteogenic switch of VSMCs. Runx2 is targeted directly by an important signaling pathway known as the canonical Wnt signaling cascade, which is known to activate the gene and control the development of bone during the period of embryogenesis. It also regulates direct bone turnover as well as bone remodeling [9]. In the canonical (β-catenin dependent) Wnt signaling pathway, the ligands bind to a specific cell membrane receptor known as the Frizzled (Fz) receptor. This binding enhances nuclear translocation of β-catenin from the cytoplasm inducing the transcription of target genes. Runx2 is one of the target genes of the Wnt signaling pathway, and stimulation of Runx2 by Wnt activates osteoblast differentiation and the formation of bone [10].

Runx2 is a chief transcription factor that is critical for ectopic blood vessel calcification. While Runx2 is not normally expressed in the aortic valves and blood vessels, several studies have reported that de novo Runx2 expression is associated with the formation of cartilaginous and calcified bone-like lesions in atherosclerotic plaques, aortic valve disease, and diabetic calcifying lesions [11].

Runx2 regulates the expression of both alkaline phosphatase (ALP) and osteocalcin genes via binding to the osteoblast-specific cis-acting element, found in the promoter regions of both genes. This may clarify the necessity for Runx2 as a molecular switch for osteogenic differentiation. Vascular cells normally exhibit low expression of Runx2, which is upregulated in atherosclerotic calcified lesions. Runx2 expression is improperly regulated by diabetes mellitus illness, which worsens aortic stiffness [12].

There is strong evidence that the RANKL/RANK/OPG signaling axis is the main key to VC. Receptor activators of NF-kB ligand (RANKL), and osteoprotegerin (OPG) are the main components of this signaling system. RANKL is a homotrimeric protein that is produced by preosteoblasts, osteoblasts, osteocytes, periosteal cells, and some other cells like activated T cells [13]. When RANKL binds to RANK, it forms a homotrimer, which triggers stimulation of nuclear factor kappa-B (NF-κB) and its nuclear translocation, which in turn increases osteogenesis gene production. In contrast, osteoprotegerin (OPG) is a glycoprotein that belongs to the family of TNF receptors. It is also called TNF receptor superfamily member 11b (TNFRS11B). OPG hinders the binding of RANKL and RANK by serving as a decoy receptor for RANK [14, 15].

A substantial body of evidence revealed that epigenetic modifications are involved in VC development and progress [16]. In the epigenetic hallmarks, microRNAs (miRNAs), are a rising class of endogenous, small noncoding single-stranded RNAs (with an average of 22 nucleotides in length). miRNAs mediate epigenetic regulation through the regulation of gene expression, either by degrading mRNAs or by inhibiting their translation; thus, miRNAs may be attractive biomarkers that will be useful in clinical practice [17, 18].

Previous studies propose that microRNAs play a bio-vital role in VSMCs trans-differentiation. However, the influence of miR-433-3p is not well documented [19]. Thus, we aimed to explore the role of serum miR-433-3p in vascular calcification in type-2 diabetes mellitus.

## Materials and methods

### Patients and samples collection

The present research was carried out at Tanta University in the Internal Medicine Department, Endocrinology and Diabetes Unit. The study included 40 patients with type 2 diabetes mellitus who were divided into two groups: 20 diabetic patients without VC (11 females and 9 males) and 20 diabetic patients with VC (10 females and 10 males). Twenty healthy subjects taken as the control group (8 females and 12 males) were also included. The American Diabetes Association's criteria were used to diagnose type 2 diabetes mellitus [20]. Diabetic patients were under treatment by either oral hypoglycemic drugs or insulin and were included in the Endocrinology and Diabetes Outpatient Clinic at Tanta University Hospital.

#### Inclusion criteria

Age of 40–63 years, 40 patients with type 2 diabetes, 20 patients of them having VC detected either by multi-slice CT coronary calcium scoring, non-contrast CT images of the aorta and both lower limbs or valvular calcification detected by echocardiography, the other 20 diabetic patients and the control non-diabetic group are free of VC detected by the same methods of screening.

#### Exclusion criteria

The current study excluded participants who were using medications like glucocorticoids, immunosuppressive agents, or cytotoxic agents, or who had a chronic illness other than diabetes, such as congestive heart failure, end-stage renal disease, liver failure, or cancer.

### Ethical statement

An informed consent was obtained prior to the start of the participation. The study protocol (which adhered to the principles of the Declaration of Helsinki II) was accepted by The Local Research Ethics Committee of Faculty of Medicine, Tanta University (Approval code: 35904/10/22).

### Clinical evaluation

Patients enrolled in this study underwent a clinical history and physical examination. This involved age, family history of diabetes, duration of diabetes, and symptoms suggestive of DM. Symptoms of chest pain, dyspnea, or intermittent claudication were also inquired.

All the participants were submitted to multi-slice CT coronary calcium scoring for detection of coronary calcification, non-contrast CT images of the aorta and both lower limbs for detection of aortic and peripheral arterial calcification, and echocardiography for detection of valvular calcification.

### Biochemical analysis

All subjects had overnight fasting blood samples taken under strict aseptic conditions. Samples were split into two portions, one of which was used to collect serum in plain tubes to measure fasting blood glucose (FBG), triglycerides (TG), total cholesterol (TC), and high-density lipoprotein cholesterol (HDL-c) levels were measured using colorimetric techniques (Spinreact, Spain), and the Friedewald equation [21] was used to estimate low- density lipoprotein cholesterol (LDL-c) levels.

For microRNA extraction, the remaining portion of the collected blood sample was stored in tubes that had been treated with ethylene diamine tetra acetic acid (EDTA).

Two hours- postprandial blood sample was obtained for assay of 2 h- post-prandial blood glucose (PP.BG).

#### Enzyme-linked immunosorbent assays (ELISAs)

It was used for the measurement of serum levels of Dickkopf-1 (MyBiosource Inc. California, USA, catalog number: MBS165224), Runx2 (MyBiosource Inc. California, USA, catalog number: MBS2512456), β-catenin (Biovision, USA, catalog number: EK3381), RANKL (Boster biological technology, USA, catalog number: EK0842), OPG (Boster biological technology, USA, catalog number: EK0480) using ELISA Reader (Stat Fax 2100, Fisher Bioblock Scientific, France).

#### Quantitative analysis of miR-433-3p gene expression

Using the Gene JET RNA Purification Kit (Thermo Scientific, USA), RNA was isolated according to the manufacturer's instructions from EDTA peripheral blood samples. To evaluate the relative expression of the miR-433-3p gene using the Step One Plus real-time PCR system (Applied Biosystem, USA), reverse transcription of total RNA was conducted using Revert Aid H minus Reverse Transcriptase (Thermo Scientific, USA) to obtain cDNA to be utilized as a template. The Primer 5.0 software was used to create the primers, and they had the following sequences: miR-433-3p Forward: 5′-GGAGAAGTACGGTGAGCCTGT-3′ and Reverse: 5′-GAACACCGAGGAGCCCATCAT-3′). The housekeeping gene small nuclear RNA U6 [22] with primer sequences; Forward: 5′-CGCTTCGGCAGCACATATACTAAAAT-3′ and Reverse: 5′-CGCTTCACGAATTTGCGTGTCAT-3′. The thermal cycling settings were initial denaturation (for 10 min.) at 95 °C, 40–45 cycles of amplification of DNA denaturation (for 15 s) at 95 °C, annealing (for 30 s) at 60 °C, extension (for 30 s) at 72 °C C. For melting curve analysis, the temperature was raised at the end of the last cycle from 63 to 95 °C C. Target gene and housekeeping gene cycle threshold (Ct) values were computed, Using the 2^−ΔΔCt^ technique, the relative gene expression was assessed [23].

### Statistical analysis

The computer program SPSS (Statistical Package for the Social Science; SPSS, Chicago, USA) version 21 for Microsoft Windows, USA, was used to present and analyze the current study's statistics. The expression for the variables was mean ± SD. One-way analysis of variance (ANOVA) was used to compare statistical differences between variables, and post hoc analysis was done afterward. In order to identify the association between various characteristics, the Pearson correlation was used. With related factors acting as independent variables, multiple linear regression analysis was used to consider the factors impacting miR-433-3p expression. Sensitivity and specificity were eventually used to complete the analysis, and the Receiver Operating Characteristic (ROC) curve was used to identify the appropriate cutoff point. If p < 0.05 statistical significance was considered to exist.

## Results

### Demographic and clinical characteristics

The demographic and clinical results of all subjects are presented in Table 1. No significant differences in age were found among all studied groups. However, a significant difference was found in the duration of diabetes mellitus between the two diabetic groups. Moreover, BMI, SBP and DBP were significantly different among groups. Furthermore, there were significant differences in FBG, Pp.BG, HbA1c, A/C ratio, lipid profile, blood urea, and serum creatinine were among the studied groups.

**Table 1:** Clinical and laboratory data of the studied groups

Variable	Control (n = 20)	Diabetic patients without VC (n = 20)	Diabetic patients with VC (n = 20)	P value
Age (years)	50.2 ± 4.6	53.6 ± 4.5	52.9 ± 6.5	0.12
BMI (kg/m^2^)	24.7 ± 2.7	35.7 ± 2.5 ^*^	38.7 ± 2.2^*#^	< 0.001*
SBP (mm hg)	121.2 ± 8.9	133.4 ± 9.3 ^*^	140.5 ± 10.9^*^	0.017*
DBP (mm hg)	76.5 ± 6.9	89.8 ± 13.6^*^	92.7 ± 14.8^*^	0.03*
Duration (years)	–	12.9 + 3.9^*^	16.5 + 3.9^*#^	< 0.05*
FBG (mg/dl)	92.4 ± 8.5	144.9 ± 6.7^*^	177.5 ± 7.6^*#^	< 0.001*
Pp. BG (mg/dl)	162.15 ± 9.4	279.9 ± 15.4^*^	320 ± 15.3^*#^	< 0.001*
HbA1c (%)	5.6 ± 0.7	7.7 ± 0.5^*^	9.2 ± 0.5^*#^	< 0.001*
A/C ratio	21.9 ± 4.3	141.5 ± 5.8^*^	183.9 ± 44^*#^	0.002*
TAG (mg/dl)	136.3 ± 4.7	172.4 ± 5.2^*^	198.1 ± 9.3^*#^	< 0.001*
TC (mg/dl)	211.4 ± 5.4	228.9 ± 3.3^*^	245.5 ± 7.1^*#^	< 0.05*
HDL-c(mg/dl)	48.4 ± 1.2	44.4 ± 0.7^*^	41.5 ± 0.6^*#^	< 0.05*
LDL-c (mg/dl)	135.3 ± 4.8	149.8 ± 4.04^*^	164.4 ± 8.1^*#^	< 0.001*
Blood urea (mg/dl)	25.9 ± 4.4	47.18 ± 3.08^*^	44.5 ± 2.7^*#^	0.02*
S. creatinine (mg/dl)	0.81 ± 0.12	1.7 ± 0.11^*^	1.9 ± 0.06^*#^	< 0.001*
Medication, n (%):				
Oral hypoglycemic drugs	0 (0%)	14 (70%)	10 (50%)	< 0.001*
Insulin	0 (0%)	4(20%)	7 (35%)	< 0.001*
Insulin + Oral hypoglycemic drugs	0 (0%)	2 (10%)	3 (15%)	< 0.05*
Statins	0 (0%)	15 (75%)	18 (90%)	< 0.001*
Aspirin	0 (0%)	19 (95%)	20 (100%)	< 0.001*

Data presented as mean + SD or n (%). P value was calculated by one-way ANOVA test followed by Tukey’s post hoc test, SPSS computer program

*n* number of cases/participants; *VC* vascular calcification; *FBG* fasting blood glucose; *Pp. BG* 2h- post prandial blood glucose; *HbA1c* glycated hemoglobin; *TAG* triacylglycerol; *TC* total cholesterol; *HDL-c* high-density lipoprotein–cholesterol; *LDL-c* low-density lipoprotein–cholesterol

*Significant difference vs. control group (P < 0.05). # Significant difference vs. diabetic patients without VC (*P < 0.05 is significant)

FBG, Pp. BG and HbA1c% levels, A/C ratio, TC, TG, and LDL-c levels were significantly increased in diabetic groups (with a significant decrease in HDL-c level) as compared with control subjects. Diabetic patients showed a significant increase in SBP and DBP in comparison with control subjects. A statistically significant difference concerning blood urea and serum creatinine levels was also detected among the studied groups.

All patients were kept on the current clinical recommendations, including diet control, lifestyle modification and medical treatment by oral hypoglycemic drugs or insulin, aspirin and statins. In diabetic patients without VC, oral hypoglycemic drugs were prescribed in 14 (70%) while 4 (20%) of patients were treated with insulin and only 2 (10%) were treated with both insulin and oral hypoglycemic drugs. In diabetic patients with VC, 10 (50%) of patients were maintained on oral hypoglycemic drugs and 7 (35%) of patients were treated with insulin but both insulin and oral hypoglycemic drugs were prescribed in 3(15%). Statins were used in 15 (75%) of diabetic patients without VC and in 18 (90%) of those with VC. In addition, 19 (95%) of diabetic patients without VC was maintained on aspirin while all case in the VC group used aspirin.

### Biochemical results

**Fig. 1:**
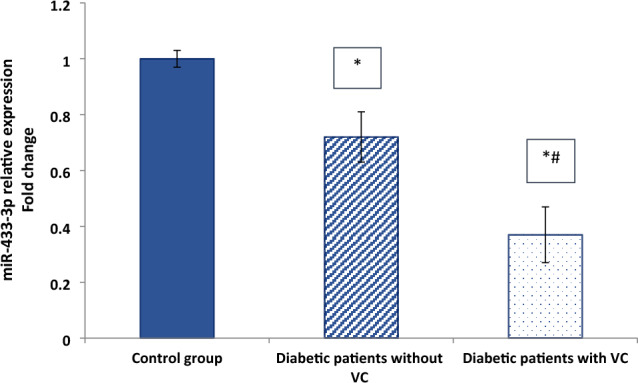
miR-433-3p relative expression in the studied groups. Data are represented as mean ± SD. *Significant difference vs. control group (P < 0.05). # Significant difference vs. diabetic patients without VC (*P < 0.05 is significant)

In comparison to the control group, the mean values of Runx2, β-catenin, and RANKL were significantly greater in the two diabetes groups, with significantly higher levels found in diabetic patients with VC. However, there were significant differences between the examined groups regarding relative miR-433-3p expression, DKK1, and OPG levels, with significantly lower values reported in the diabetic groups compared to the control group as presented in (Table 2 and Fig. 1).

**Table 2:** Biochemical and molecular results of the studied groups

Variable	Control (n = 20)	Diabetic patients without VC (n = 20)	Diabetic patients with VC (n = 20)	P value
Runx2 (ng/ml)	5.9 ± 0.46	11.4 ± 1.11^*^	16.5 ± 0.66^*^	< 0.001*
DKK1 (ng/ml)	119.7 ± 3.88	98 ± 4.3^*^	84.5 ± 3.8^*#^	0.004*
β-catenin (ng/ml)	1.73 ± 0.35	4.7 ± 0.45^*^	8.8 ± 0.25^*#^	< 0.001*
RANKL (pg/ml)	2411 ± 173.5	3113 ± 302.6^*^	4020.2 ± 106.2^*#^	< 0.001*
*OPG (*pg/ml)	766.6 ± 64.9	547.6 ± 26.3^*^	412.7 ± 87.7^*#^	< 0.05*

Data presented as mean + SD. P value was calculated by one-way ANOVA test followed by Tukey’s post hoc test, SPSS computer program

*n* number of cases/participants; *VC,*vascular calcification; *RUNX2* runt-related transcription factor 2; *DKK1* dickkopf-1; *RANKL* receptor activator of nuclear factor kappa-Β ligand; *OPG* osteoprotegerin

*Significant difference vs. control group (P < 0.05). # Significant difference vs. diabetic patients without VC (*P < 0.05 is significant)

### Correlations between the studied parameters

A significant negative correlation was detected between miR-433-3p relative expression and Runx2, β-catenin, RANKL, FBG, and Pp.BG, TC, TG, blood urea and serum creatinine, HbA1c, and A/C ratio. While miR-433-3p expression level showed a significant positive correlation with DKK1 and OPG levels. (Table 3).

**Table 3:** Correlation between the studied parameters

	DKK1		Runx2		β-catenin		*RANKL*		*OPG*		*miR-433-3p*	
	r	P value	r	P value	r	P value	r	P value	r	P value	r	P value
DKK1												
Runx2	0.568-	< 0.001*										
β-catenin	0.935-	< 0.001*	0.523	< 0.001*								
RANKL	0.892-	< 0.001*	0.394	< 0.001*	0.958	< 0.001*						
OPG	0.882	< 0.001*	0.551-	< 0.001*	0.893-	< 0.001*	0.870-	< 0.001*				
miR-433-3p	0.941	< 0.001*	0.536-	< 0.001*	0.969-	< 0.001*	0.935-	< 0.001*	0.9	< 0.001*		
FBG	0.935-	< 0.001*	0.599	< 0.001*	0.952	< 0.001*	0.903	< 0.001*	0.877-	< 0.001*	0.933-	< 0.001*
Pp. BG	0.941-	< 0.001*	0.573	< 0.001*	0.910	< 0.001*	0.880	< 0.001*	0.872-	< 0.001*	0.915-	< 0.001*
Duration	0.883-	< 0.001*	0.614	< 0.001*	0.835	< 0.001*	0.776	< 0.001*	0.821-	< 0.001*	0.850-	< 0.001*
HbA1c	0.892-	< 0.001*	0.506	< 0.001*	0.913	< 0.001*	0.903	< 0.001*	0.875-	< 0.001*	0.872-	< 0.001*
A/C ratio	0.875-	< 0.001*	0.518	< 0.001*	0.861	< 0.001*	0.828	< 0.001*	0.797-	< 0.001*	0.849-	< 0.001*
TAG	0.938-	< 0.001*	0.519	< 0.001*	0.946	< 0.001*	0.915	< 0.001*	0.884-	< 0.001*	0.941-	< 0.001*
TC	0.898-	< 0.001*	0.533	< 0.001*	0.931	< 0.001*	0.871	< 0.001*	0.846-	< 0.001*	0.920-	< 0.001*
Blood urea	0.819-	< 0.001*	0.570	< 0.001*	0.714	< 0.001*	0.707	< 0.001*	0.786-	< 0.001*	0.752-	< 0.001*
S. creatinine	0.932-	< 0.001*	0.56	< 0.001*	0.873	< 0.001*	0.843	< 0.001*	0.863-	< 0.001*	0.880-	< 0.001*

*DKK1* dickkopf-1; *RUNX2* runt-related transcription factor 2; *RANKL* receptor activator of nuclear factor kappa-Β ligand; *OPG* osteoprotegerin; *miR-433-3p* microRNA-433-3p, *HbA1c* glycated hemoglobin; r, Pearson’s correlation coefficient. (*P < 0.05 is significant)

### Multiple linear regression analysis of miR-433-3p -related factors

HbA1c was considered as the dependent variable while the other studied parameters were considered as independent variables. miR-433-3p relative expression was revealed to be the independent predictor for VC (B 2.919, P value < 0.001*) (Table 4).

**Table 4:** Multiple linear regression analysis for potential predictors of vascular calcification in type 2 diabetic patients

Variable	Unstandardized coeffecients		Standardized coeffecients		
	B	Standard error	Beta	t	P value
*miR-433-3p*	2.919	0.953	0.648	3.06	< 0.001*
DKK1	− 0.037	0.016	− 0.356	− 2.4	0.02*
Runx2	0.012	0.013	0.16	1.927	0.05
β-catenin	0.282	0.135	0.524	2.081	0.042*
*RANKL*	0.001	0.000	0.437	2.348	0.023*
*OPG*	− 0.003	0.001	− 0.263	− 2.307	0.025*

Dependent variable: HbA1c %

*miR-433-3p* microRNA-433-3p, *DKK1* Dickkopf-1; *RUNX2* runt-related transcription factor 2; *RANKL* receptor activator of nuclear factor kappa-Β ligand; *OPGL* osteoprotegerin; *HbA1c* glycated hemoglobin

### ROC curve of relative miR-433-3p expression as an early marker for discriminating diabetic patients with VC from healthy controls and diabetic patients without VC

**Fig. 2:**
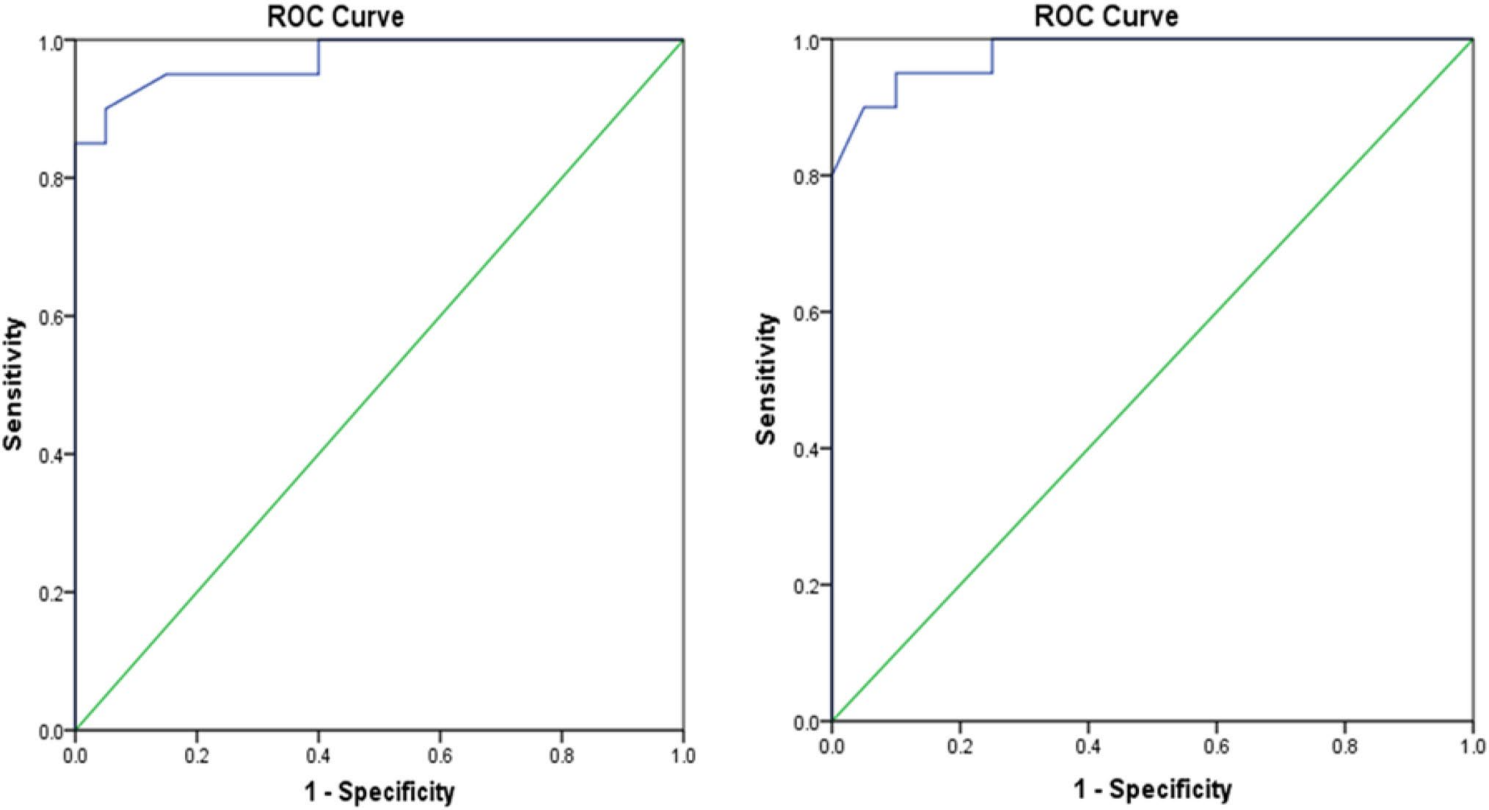
ROC curve of miR-433-3p expression for discriminating **A**: Diabetic patients with VC from healthy controls; **B**: Diabetic patients with VC from diabetic patients without VC

ROC curve was used to measure miR-433-3p relative expression value for discriminating diabetic patients with VC from healthy controls (Fig. 2A) where the optimal cut-off point was 0.93 with a sensitivity of 95% and specificity of 92%. The area under the curve was 0.973. In (Fig. 2B) the ROC curve was applied to discriminate diabetic patients with VC from those without VC where the optimal cut-off point was 0.72 with a sensitivity of 95% and specificity of 95%. The area under the curve was 0.98.

## Discussion

Vascular calcification (VC) is highly prevalent in patients with type-2 diabetes, chronic kidney disease, and hypertension, leading to an augmented risk of cardiovascular morbidity and mortality [24]. Numerous molecular processes and signaling pathways, including aberrant calcium or phosphate deposition, osteogenic trans-differentiation of vascular smooth muscle cells (VSMCs), oxidative stress, inflammation, and apoptosis, have been identified as contributing to its pathogenesis. [25].

At present, researchers pay attention to the imminent role of microRNAs (miRNAs) as newly revealed key factors involved in the pathogenesis of a wide variety of diseases. miRNAs are classes of non-coding small RNAs that can regulate numerous target genes post-transcriptionally. Consequently, miRNA-based regulation has been recognized as a prospective possibility for researching disease genesis and treating several disorders at the epigenetic level [26].

According to Goettsch et al., miRNAs are highly stable and found in blood (plasma, platelets, erythrocytes, and nucleated blood cells) due to their encapsulation in extracellular vesicles, association with a protein complex containing the RNA-binding protein Argonaute 2, or inclusion in lipoprotein complexes. These associations protect the miRNAs from degrading while they are in circulation [27].

In CVD patients, altered miRNA expression has been detected, and certain miRNAs have been documented to be involved in multiple cardiovascular diseases, such as atherosclerosis, VC, arrhythmias, and myocardial infarction [28]. A variety of miRNAs have been involved in the pathogenesis of vascular calcification, whereas others may have a protective role. Therefore, miRNA might be effective targets for preventing VC and its detrimental consequences [29].

In our study herein, we reported the role of miR-433-3p in VC for the first time. We identified miR-433-3p as a negative regulator of VSMC calcification. However, other research studies its role in the trans-differentiation of VSMCs and atherosclerosis [30, 31].

Our data revealed that a statistically significant down-regulation of miR-433-3p was noted among both diabetic groups with and without VC compared to each other and the control group, with profound downregulation in those with VC. The ROC curve analysis displayed that the miR-433-3p relative expression could discriminate diabetic patients with VC from healthy control with high levels of accuracy (AUC = 0.973). Also, it has a significant ability to discriminate diabetic patients with VC from those without VC patients (AUC = 0.98).

Likewise, Mir et al. [32] found that miR-433-3p was down-regulated in patients with obesity and metabolic syndrome and negatively correlated with HbA1c, suggesting its pathophysiologic role in various metabolic diseases like diabetes. Similarly, miR-433-3p was overexpressed in healthy individuals compared to diabetic nephropathy patients, ensuring miR-433-3p’s protective role in diabetic conditions [33]

The miR-433-3p level was decreased in women after gestational diabetes mellitus diagnosis, according to Sørensen et al. [34]. The miR-433-3p was down-regulated in insulin-secreting MIN6 cells in a high glucose environment in vitro; however, the β-cells were protected by restoring miR-433-3p levels using miR-mimics. In an effort to maintain glucose homeostasis, it is conceivable that miR-433-3p could contribute to the compensatory effects [34].

It was also reported that low levels of miR-433-3p were associated with a higher risk of type 2 diabetes seen in PCOS women since they are thought to protect pancreatic β-cells from glucose toxicity [35].

Likewise, previous studies demonstrated that miR-433-3p directly targets cyclooxygenase 2 to protect mouse pancreatic cells from the high glucose-induced decrease of cell viability. Its expression was shown to be down-regulated in high glucose, and miR-433 mimic transfection significantly increased its expression, which significantly increased cell viability and proliferation in high glucose conditions by suppressing apoptosis and accelerating cell cycle progression, suggesting its pathophysiologic role in various metabolic diseases including diabetes [36].

According to Li et al., miR-433 plays a significant role in TGF-β/Smad3-induced renal fibrosis by creating a positive feedback loop that amplifies TGF-β/Smad3 signaling. This suggests that it could be a promising therapeutic target for tissue fibrosis [37].

Moreover, miR-433-3p was found to be expressed in the osteoblast lineage but not in osteoclasts. A single nucleotide polymorphism in human osteonectin, a plentiful non-collagenous bone matrix protein that controls osteoblast development and survival, was discovered to be variably targeted by miR-433-3p [38].

Notably, miR-433-3p is progressively reduced during differentiation of osteoblasts from primary mouse bone marrow stromal cells in vitro. It was detected to target Runx2 and also to be inhibited by bone morphogenetic protein (BMP) signaling. Similarly, miR-433-3p targets R-spondin 3 (Rspo3), a leucine-rich repeat-containing G-protein coupled receptor (LGR) ligand that augments WNT signaling. Particularly, WNT canonical signaling is also suppressed by the activity of miR-433-3p [39].

In this context, it was recognized that miR-433-3p was shown to be reduced during BMP2-induced osteoblastic differentiation of C3H10T1/2 cells and to target the Runx2 3′ UTR [40].

Runx2, also known as AML3 or Cbfa1, is an important transcription factor that is necessary for skeletal formation and remodeling. It is the chief regulator of bone formation. It is up-regulated within calcifying VSMCs and may be responsible for this trans-development; it is also important for osteoblast differentiation [41]. Lin et al. demonstrated that SMC-specific Runx2 has an imminent role in both osteoblastic differentiation and chondrocyte maturation during atherosclerosis-induced VC [42].

Unsurprisingly, our results supported earlier research [43, 44] and validated that the Runx2 level was significantly greater in diabetic groups with and without VC compared to the control group with a higher level demonstrated in those with VC.

Emerging evidence testifies the involvement of the canonical WNT signaling pathway in the pathogenesis of VC as WNT/β-catenin signaling is essential for the osteogenic differentiation of pluripotent mesenchymal cells. The extracellular signal, membrane segment, cytoplasmic segment, and nuclear segment are the four segments that make up the Wnt/β-catenin pathway. Wnt proteins, including Wnt3a, Wnt1, and Wnt5a, play a major role in the transmission of extracellular signals. The Wnt signaling pathway, which is made up of a large part of the β-catenin protein, is crucial for cell signaling [45].

The phosphorylation of β-catenin was brought on by the lack of Wnt ligands. The fragments of the broken-down complex cannot be bonded to or spread out by the phosphorylated β-catenin. Because β-catenin cannot be degraded, it accumulates within cells and is subsequently released into the extracellular matrix. Studies on β-catenin focus mostly on its accumulation within the cell and its transcription into the nucleus [46].

Recently, a small number of instances have proven that β-catenin can be found in serum and may be related to the progression of type 2 diabetes, PTEN hamartoma tumor syndrome, early-onset ulcerative colitis, hepatitis C, and hepatitis B. These results revealed that serum disease-associated indicators may be identified by β-catenin [47].

Runx2 is induced as a result of pro-osteogenic stimuli activating the WNT/ β -catenin signaling pathway. Runx2 then controls the production of many bone-related proteins, including sclerostin, osteocalcin, and osterix, and also can control crucial processes necessary for osteoblast development and phenotypic characterization [8, 44].

Atherosclerotic injury is the reason that triggered the heightened plasma RUNX2 level. Therefore, early arterial lesions and calcification appear to be more caused by elevated levels of RUNX2. RUNX2 is crucial for the calcification of vascular smooth muscle cells brought on by oxidative stress. This indicates that arterial injury is more affected by elevated plasma RUNX2 levels. Therefore, it appears that early artery lesions are more likely to be caused by elevated levels of RUNX2 [48].

Indeed, a notable soluble dominant antagonist of the canonical WNT protein is Dickkopf1 (DKK1). It was the first and most characteristic molecule to bind to the single-pass trans-membrane receptor proteins Kremen 1 and Kremen 2, as well as to the plasma membrane-located WNT co-receptors LRP5 and LRP6 to suppress canonical WNT signaling [49].

In a previous study, DKK1 concentration was inversely co-related to aortic calcification and coronary artery disease [50]. Likewise, our results revealed that the two diabetes groups, particularly diabetic patients with VC, showed a considerable rise in β-catenin levels together with a concurrent decline in DDK1. In agreement with our finding, Tang et al. proved the vital role of miR-433-3p in the DKK1/WNT/β-catenin signaling pathway by reducing DKK1 expression and enhancing osteoblasts differentiation [51].

In accordance with our results, RANKL/RANK/OPG axis dysregulation appears to be involved in the progression of VC [52]. It appears to be the final effector of many osteotropic factors. It consists of the transmembrane protein RANK, its ligand (RANKL), and the soluble receptor OPG that binds to the RANKL, thereby interfering with the RANK-RANKL interaction [5, 53].

Bucay et al. reported that OPG knock-out (OPG^−/−^) mice developed osteoporosis and massive arterial calcification [54]. Additionally, RANKL expression was up-regulated in calcified arteries [55]. RANKL directly promotes the calcification of vascular smooth muscle cells by binding to RANK and promoting BMP4 production via the alternative NF-B pathway. RANKL also acts indirectly by raising macrophage paracrine pro-calcific activity by promoting the release of tumor necrosis factor-alpha and interleukin-6 [56].

Nie et al. showed that the WNT/ β-catenin pathway activation significantly promoted the calcification and up-regulated RANKL gene expression. Furthermore, WNT/β-catenin pathway activity had a positive relation with the degree of arterial or cell calcification. An active WNT/-catenin pathway during calcification increased the degree of calcification, especially in the early stage, which was likely induced by increased expression of the proteins RANKL, WNT3a, and WNT7a [57].

In agreement with this premise, Motovska et al. showed a connection between the RANKL/RANK/OPG axis, WNT/DKK-1 signaling, and the onset of atherosclerosis. These findings suggest that these factors may be involved in the regulation of VC in calcified aortic stenosis [5].

## Conclusion

Our study validated, for the first time the protective role of miR-433-3p against the development of VC, offering an innovative guard against the common mechanisms of VC pathogenesis via targeting of the WNT/β-Catenin and RANKL/RANK/OPG signaling pathways. The data presented here also suggest that miR-433-3p could be considered a biomarker useful to support the diagnosis and prediction of VC.

## Limitation

The current study has some limitations. First, a small number of patients in this study is an important limitation, so a large number of patients is required to validate our findings. Also, this study is based on a single center, which does not reflect the whole population. Thus, multicenter studies are required to validate these results.

For better data validation, silence/overexpress miR-433-3p in VSCMs and its effect on osteogenic differentiation genes and VC should be studied in the upcoming research.

## Data Availability

It is available upon reasonable request**.**

## Abbreviations

**ALP**: Alkaline phosphatase
**ANOVA**: One-way analysis of variance
**BMI**: Body mass index
**BMP**: Bone morphogenetic protein
**CVD**: Cardiovascular diseases
**DKK1**: Dickkopf-1
**DM**: Diabetes mellitus
**ELISA**: Enzyme-linked immunosorbent assay
**FBG**: Fasting blood glucose
**HDL-c**: High- density lipoprotein cholesterol
**LDL-c**: Low- density lipoprotein cholesterol
**miR-433-3p**: MicroRNA- 433-3p
**NF-κB**: Nuclear factor kappa-B
**OPG**: Osteoprotegerin
**RANKL**: Receptor activator of nuclear factor kappa-B ligand
**ROC**: Receiver Operating Characteristic
**Runx2**: Runt-related transcription factor 2
**TC**: Total cholesterol
**TG**: Triglycerides
**TNF**: Tumor necrosis factor
**UACR**: Urinary albumin/creatinine ratio
**VC**: Vascular calcification
**VSMCs**: Vascular smooth muscle cells
